# Cochlear implantation without general anaesthesia: a resource-efficient surgical pathway

**DOI:** 10.1017/S002221512610543X

**Published:** 2026-08

**Authors:** Holly Mould, Emma Stapleton

**Affiliations:** 1 University of Manchester Institute of Science and Technology Department of Biomo, UK; 2 Manchester Academic Health Sciences Centre, The University of Manchester Faculty of Biology Medicine and Health, UK; 3 Department of Otolaryngology, Manchester Royal Infirmary, UK

**Keywords:** carbon footprint, cochlear implantation, healthcare costs, local anaesthesia, sustainability

## Abstract

**Objectives:**

This study aimed to compare the environmental and financial impact of cochlear implantation performed with and without general anaesthesia.

**Methods:**

Two cochlear implant procedures performed on the same day by the same surgical and anaesthetic teams were prospectively evaluated: one under total intravenous anaesthesia with bispectral index monitoring, and one under local anaesthesia with propofol sedation. Anaesthetic drugs, consumables, equipment, electricity use and procedural duration were recorded. Financial costs were estimated using procurement data and national formularies. Carbon dioxide equivalent emissions were calculated using published conversion factors.

**Results:**

Local anaesthesia was associated with lower cost (£59 vs. £179) and lower carbon footprint (2.85 vs. 5.28 kg carbon dioxide equivalent). Procedural duration was similar between pathways (71 vs. 74 minutes).

**Conclusion:**

Cochlear implantation without general anaesthesia may reduce environmental and financial cost without prolonging procedural duration. Anaesthetic pathway selection represents a modifiable determinant of resource use in otological practice.

## Introduction

Cochlear implantation is conventionally performed under general anaesthesia, although local anaesthesia with sedation is increasingly used in selected patients. This approach may offer clinical advantages, including avoidance of airway instrumentation and reduced physiological stress, particularly in patients at higher anaesthetic risk.

Alongside these clinical considerations, healthcare systems face increasing pressure to improve efficiency and reduce environmental impact. The National Health Service contributes substantially to greenhouse gas emissions, and anaesthetic practice is a recognised contributor to this footprint.^1^^,^^2^ Despite this, environmental and financial implications are rarely incorporated into peri-operative decision-making.^3^^,^^4^ Emerging sustainable otology initiatives, including the “Green Cochlea” paradigm, have highlighted multiple opportunities to reduce the environmental impact of hearing implant care, including anaesthetic practice.^5^

Where more than one anaesthetic approach is feasible, the choice of technique represents not only a clinical decision but also a resource pathway with measurable consequences. Cochlear implantation provides a useful model for comparison, as both general and local anaesthetic techniques are viable in routine practice.^6^

This report compares the environmental and financial impact of cochlear implantation performed with and without general anaesthesia.

## Short Communication

Two cochlear implant procedures were evaluated prospectively as part of a service evaluation using non-identifiable peri-operative data. Both procedures were performed on the same day by the same surgical and anaesthetic teams. One procedure was undertaken under total intravenous anaesthesia (TIVA) with bispectral index monitoring, and the other under local anaesthesia with propofol sedation.

All anaesthetic drugs, consumables and equipment used during each procedure were recorded. Electrical equipment usage was documented, including duration of use and power requirements.

Financial costs were estimated using institutional procurement data for consumables and national formularies for drug pricing. Carbon dioxide equivalent (CO₂e) emissions were calculated using published conversion factors, incorporating drug production, equipment manufacture, waste disposal (assuming incineration) and electricity generation.^3^

The analysis was limited to anaesthetic-related resource use; surgical equipment and staffing were equivalent between procedures and were not included. Broader pathway factors including recovery resource utilisation, organisational costs and longer-term service impacts were not assessed.

Total procedural duration, measured from arrival in the anaesthetic room to departure from theatre and including anaesthetic administration and theatre setup, was similar between the two pathways (74 minutes for general anaesthesia and 71 minutes for local anaesthesia with sedation).

Local anaesthesia was associated with lower overall cost (£59 vs. £179) and lower carbon footprint (2.85 vs. 5.28 kg CO₂e).

The principal contributors to increased cost in the general anaesthesia pathway were tracheal intubation equipment and facial nerve monitoring. Increased carbon emissions were driven by equipment production and disposal, and higher electricity consumption associated with anaesthetic machinery.

Key differences between pathways are summarised in Table 1.

**Table 1.** Comparison of anaesthetic pathways in cochlear implantation Table 1 long description.

Parameter	General anaesthesia	Local anaesthesia with sedation
Cost (£)	179	59
CO₂e (kg)	5.28	2.85
Airway management	Tracheal intubation	Spontaneous ventilation
Facial nerve monitoring	Used	Not used
Equipment use	Higher	Lower
Electricity consumption	Higher	Lower
Total procedural duration (min)	74	71

CO₂e = carbon dioxide equivalent.

## Discussion

This report demonstrates that cochlear implantation performed without general anaesthesia is associated with reduced environmental and financial cost. While anaesthetic technique is traditionally considered in terms of patient safety and peri-operative outcomes, these findings highlight its additional role as a determinant of resource use.

The observed differences were driven primarily by equipment utilisation and energy consumption rather than drug costs alone. Avoidance of airway instrumentation and neuromonitoring reduced both consumable use and waste generation, while reduced reliance on anaesthetic machinery lowered electricity consumption.

Although the absolute differences per case are modest, the cumulative effect across a high-volume procedure such as cochlear implantation may be considerable. Small reductions in resource use at the level of individual cases may translate into meaningful system-level savings when applied across a programme.

In current practice, environmental and financial considerations are rarely visible at the point of care. Where more than one anaesthetic approach is clinically appropriate, incorporating resource considerations into decision-making may represent an opportunity for incremental improvement.^4^

Local anaesthesia is not suitable for all patients and requires appropriate expertise and patient selection. Recognised challenges include drilling-related noise and vibration, caloric responses, patient movement and communication with the awake patient. Practical strategies to overcome these challenges have been described previously and may facilitate wider adoption where appropriate pathways and expertise exist.^6^^,^^7^

The present analysis was intentionally restricted to anaesthetic-related resource utilisation and should not be interpreted as a complete economic or environmental assessment of the cochlear implantation pathway. Factors such as recovery resource utilisation, organisational costs and wider service delivery considerations may also influence overall impact and warrant evaluation in future studies.

This study is limited by its small sample size and reliance on estimated emission data. Nevertheless, it demonstrates the feasibility of capturing environmental and financial metrics alongside routine clinical care and highlights consistent differences between anaesthetic pathways.

## Table 1 Long description

Comparison of two anaesthetic pathways for cochlear implantation across cost, carbon emissions, airway approach, monitoring, resource use, and procedure duration. General anaesthesia costs 179 pounds versus 59 pounds for local anaesthesia with sedation. Carbon emissions are higher with general anaesthesia at 5.28 kilograms of carbon dioxide equivalent compared with 2.85 kilograms for local anaesthesia with sedation. Airway management differs: general anaesthesia uses tracheal intubation, while local anaesthesia with sedation uses spontaneous ventilation. Facial nerve monitoring is used with general anaesthesia and not used with local anaesthesia with sedation. Equipment use and electricity consumption are both higher for general anaesthesia and lower for local anaesthesia with sedation. Total procedural duration is similar, at 74 minutes for general anaesthesia and 71 minutes for local anaesthesia with sedation. Values reflect the listed parameters only and do not capture differences in patient selection, safety outcomes, or downstream impacts.

Navigate back to Table 1.
